# Association between Interleukin-10 Gene Polymorphisms and Behcet's Disease Susceptibility: Evidence from a Meta-Analysis

**DOI:** 10.1155/2020/9382609

**Published:** 2020-06-19

**Authors:** Xue-Feng Li, Zhi-Zhen Huang, Yuan-Yuan Hu, Yu-Ming Niu, Jun-Wei Cai

**Affiliations:** ^1^Department of Endocrinology, Hubei Key Laboratory of Embryonic Stem Cell Research, Taihe Hospital, Hubei University of Medicine, Shiyan 442000, China; ^2^Department of Stomatology, Evidence-Based Medicine and Clinical Research, Hubei Key Laboratory of Embryonic Stem Cell Research, Taihe Hospital, Hubei University of Medicine, Shiyan 442000, China

## Abstract

Epidemiological studies have demonstrated that interleukin-10 (IL-10) polymorphisms may be associated with the development of Behcet's disease (BD). However, the published results were inconsistent. Therefore, this meta-analysis was conducted to derive a more precise relationship between IL-10 polymorphisms and BD susceptibility. Online databases (PubMed, Embase, Science Citation Index (SCI), CNKI, and WanFang) were searched to identify eligible studies. Odds ratio (OR) and a 95% confidence interval (CI) were applied to assess the relationship strength between IL-10 -1082A>G (rs1800896), -819T>C (rs1800871), and -592A>C (rs1800872) polymorphisms and BD susceptibility. Publication bias, sensitivity, and cumulative analyses were conducted to measure the robustness of our findings. Finally, fifteen articles (36 independent case-control studies) involving 5,971 patients and 8,913 controls were included. Overall, significant associations between -819T>C polymorphisms and BD risk were observed in the total population (C vs. T: OR = 0.72, 95%CI = 0.67‐0.77, *P* < 0.01, *I*^2^ = 36.6%; TC vs. TT: OR = 0.73, 95%CI = 0.66‐0.80, *P* < 0.01, *I*^2^ = 23.0%; CC vs. TT: OR = 0.52, 95%CI = 0.39‐0.70, *P* < 0.01, *I*^2^ = 53.7%; TC+CC vs. TT: OR = 0.67, 95%CI = 0.61‐0.71, *P* < 0.01, *I*^2^ = 22.1%; and CC vs. TT+TC: OR = 0.66, 95%CI = 0.53‐0.82, *P* < 0.01, *I*^2^ = 57.8%). Moreover, the IL-10 -592 A>C polymorphism and the ACC haplotype exhibited a significant, protective effect against BD susceptibility. In summary, our meta-analysis suggested that IL-10 gene polymorphisms may play a salient role for BD development.

## 1. Introduction

Behcet's disease (BD) is a chronic polygenic autoinflammatory disorder, characterized by multiple affected body parts, with common symptoms including recurrent oral ulcers, ocular inflammation, genital ulcers, and skin lesions [1, 2]. In addition, other organs, such as the musculoskeletal, gastrointestinal, central nervous, and pulmonary systems may be affected and present some clinical manifestations [3]. Today, the most authoritative diagnostic criteria were made according to the criteria of the International Study Group of BD: oral ulceration plus any two genital ulcerations, typical defined eye lesions, typical defined skin lesions, or a positive pathergy test [4]. BD is most common in Asian countries, such as China and Japan (East Asian (EA)), as well as in the Middle East (ME) around the regions of the Old Silk Road, such as Turkey and Iran [5]. Epidemiological studies have shown that BD has the highest prevalence in the population of Jordan, a slightly lower prevalence in East Asian countries, and the lowest prevalence in Western countries [6–9]. Pathologically, the lesions always impair arterioles, venules, and microvessels, resulting in the development of microvasculitis with vascular wall necrosis, dilation, and rupture. To date, the etiology and pathogenesis of BD have not been elucidated; however, environmental risks, immunological aberrations, and multiple genetic factors are considered risk factors that can increase susceptibility to BD [10, 11].

Interleukin-10 has been proven to be an important cytokine-suppressing, autoimmune and inflammatory response factor. IL-10 may inhibit the antigen-presenting process by downregulating the expression of HLA molecules on the surface of a cell and suppressing the expression of multiple proinflammatory cytokines, such as TNF-*α*, IL-1, IL-6, and IL-8, by inhibiting T cells. IL-10 is secreted by T lymphocytes (mainly Th2 subsets), B lymphocytes, NK cells, monocytes, and macrophages and is involved in the pathogenesis of BD [12, 13].

A number of studies have indicated that the aberrant expression of IL-10 might lead to an inflammatory state that is more susceptible to BD [14, 15]. A recent genome-wide association study (GWAS) demonstrated that a common polymorphic locus of rs1518111 in IL-10 may cause individuals to have a predisposition to BD [16]. The three most common single nucleotide polymorphisms (SNPs) were in the promoter of IL-10 with loci of -1082A>G (rs1800896), -819T>C (rs1800871), and -592A>C (rs1800872), which could influence the gene transcription and expression by changing the promoter binding site, resulting in differing susceptibility of individuals to the disease. To date, a large number of studies have been published on the association between IL-10 polymorphisms and BD susceptibility.

In 2013, Liang et al. conducted the first meta-analysis based on four case-control studies, exploring the potential association between the three IL-10 polymorphisms mentioned and BD risk [17]. In the last six years, new studies have been published on this topic, but the results are still inconsistent and remain inconclusive. Therefore, we conducted an updated meta-analysis to clarify the genetic association between the -1082A>G (rs1800896), -819T>C (rs1800871), and -592A>C (rs1800872) polymorphisms of the IL-10 gene and the risk of BD.

## 2. Materials and Methods

This meta-analysis was conducted according to the guidelines of the Preferred Reporting Items for Systematic Reviews and Meta-Analyses (PRISMA) statement [18]. All included data were based on published studies, and no ethical issues were involved.

### 2.1. Search Strategy

Three English databases (PubMed, Embase, and Science Citation Index (SCI)) and two Chinese databases (CNKI and WanFang) were searched with the following terms from their inception up to May 1, 2019: “Interleukin 10,” “IL-10,” “rs1800896,” “rs1800871,” “rs1800872,” “polymorphism,” “variant,” “mutation,” “Behcet's disease,” and “BD.” Some relevant papers cited within retrieved articles were also reviewed through manual searches. The following search strategy was used (PubMed for example):

(#1) Interleukin 10
(#2) IL-10
(#3) rs1800896
(#4) rs1800871
(#5) rs1800872
(#6) #1 OR #2 OR #3 OR #4 OR #5
(#7) Polymorphism
(#8) Variant
(#9) Mutation
(#10) #7 OR #8 OR #9
(#11) Behcet's disease
(#12) BD
(#13) #11 OR #12
(#14) #6 AND #10 AND #13.

### 2.2. Eligibility Criteria

All selected studies met the following criteria: (1) case-control design was employed; (2) some polymorphisms of the IL-10 gene was reported; (3) sufficient data on genotypes of each polymorphism locus were provided to calculate the odds ratios (ORs) and 95% confidence intervals (CIs); (4) the most recent or largest sample sizes were selected when multiple publications were repeatedly reported with the same theme; and (5) the articles were written only in English and Chinese. The studies were excluded if they were (1) letters, reviews, or conference reports; (2) duplicate reports with the same theme and samples; (3) animal models or cell line studies; or (4) studies without sufficient data.

### 2.3. Data Extraction

Two independent investigators (LXF and HZZ) reviewed the articles and extracted the following information: name of first author in each study, year of publication, study country or region where the study was conducted, ethnicity of research population, design of the control, sample sizes of patients with BD and controls, frequency genotype of distribution data, and genotyping method used. Disagreements were settled through discussion.

### 2.4. Quality Assessment

Each study included was evaluated via risk assessment of bias by two authors, LXF and HZZ, according to the modified Newcastle-Ottawa Quality Assessment Scale [19]. The score was assigned on the basis of five parameters (representativeness of cases, source of controls, Hardy-Weinberg's equilibrium (HWE) in controls, genotyping examination, and association assessment) and ranked from zero to 11 points. Studies with a score of at least 7 were considered to be of high quality (Table 1).

### 2.5. Statistical Analysis

The pooled ORs with 95% CIs were used to assess the strength of the associations between the IL-10 gene polymorphisms and BD risk. For example, the genetic models of the -1082A>G (rs1800896) polymorphisms were examined: allele contrast (G vs. A), codominant models (heterozygote model: AG vs. AA; homozygote model: GG vs. AA), dominant model (AG+GG vs. AA), and recessive model (GG vs. AA+AG). Similar genetic models were also examined with the other polymorphic loci (-819T>C (rs1800871) and -592A>C (rs1800872)). Heterogeneity was investigated by Cochran's *χ*^2^-based *Q*-statistic among the included studies [20]. A fixed-effect model (Mantel-Haenszel's method) was used to evaluate the results if heterogeneity was not significant (*I*^2^ < 40%) [21]. Otherwise, a random-effects model (DerSimonian and Laird's method) was used [22]. Subgroup analyses were performed according to HWE status, ethnic differences, and control design and genotyping methods. Cumulative analyses were conducted to test the changing trends in the results, and sensitivity analyses were conducted to test the robustness of the original results with the published studies which were added gradually. Publication bias testing was conducted with Egger's bias test and Begg's funnel plots [23, 24]. Statistical analysis was performed using STATA version 14.0 (Stata Corporation, College Station, TX, USA), and *P* values < 0.05 were considered to be significant.

## 3. Results

### 3.1. Study Characteristics

**Figure 1:**
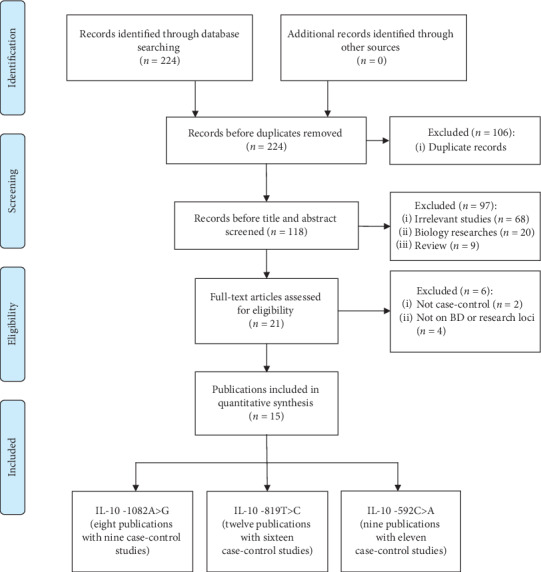
Flow diagram of the study selection process.

A total of 224 potential articles were retrieved from the databases at the first step. During the subsequent screening and selection steps of duplicate checks, title and abstract checks, and text reviews, 209 articles were excluded according to the eligibility criteria. Finally, fifteen articles (36 independent case-control studies) involving 5,971 patients and 8,913 controls were included in this meta-analysis (Figure 1) [5, 13, 25–37]. Of these studies, nine case-control studies focused on -1082A>G [5, 13, 25–28, 31, 34], sixteen case-control studies focused on -819T>C [5, 13, 25–32, 35, 36], and eleven case-control studies focused on -592A>C [13, 26–29, 31, 33, 35, 37]. In addition, ten publications came from ME [5, 25–28, 31, 33–36], five publications came from East Asia (EA) [13, 30, 32, 35], and three publications came from European descendants [25, 29, 37]. Moreover, the genotype frequencies in the control group in two studies of the -1082A>G polymorphism and one study of the -592A>C polymorphism deviated from HWE. Furthermore, four articles only had allele data, which resulted in the HWE assessment not being calculated [34–37]. All the related characteristics are presented in Table 2.

### 3.2. Quantitative Analysis

#### 3.2.1. -1082A>G Polymorphism and BD Risk

**Figure 2:**
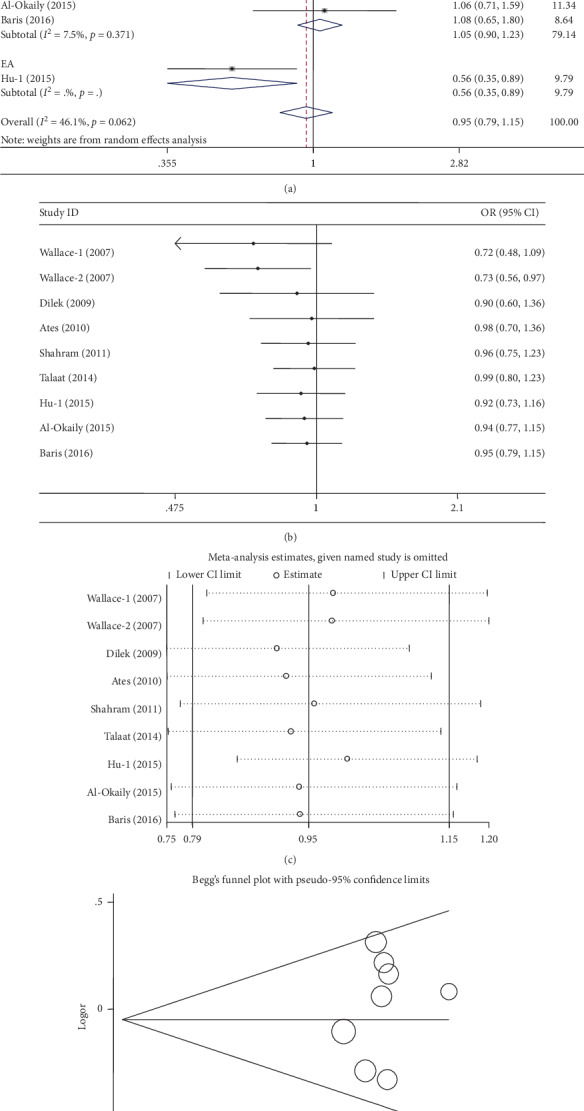
Statistical analysis of the association between IL-10 -1082A>G polymorphism and BD risk in the G vs. A model. (a) ORs and 95% CIs; (b) cumulative analysis; (c) sensitivity analysis; (d) publication bias.

Nine case-control studies, involving 1,042 patients and 1,389 controls, were identified on the association between the -1082A>G polymorphism and BD risk in this meta-analysis, and these are shown in Table 3. Overall, the synthetic data indicated that there was no significant association between the -1082A>G polymorphism and BD risk in the overall population for all five genetic models (G vs. A: OR = 0.94, 95%CI = 0.77‐1.15, *P* = 0.55, *I*^2^ = 52.0%; AG vs. AA: OR = 0.91, 95%CI = 0.65‐1.29, *P* = 0.60, *I*^2^ = 64.2%; GG vs. AA: OR = 0.89, 95%CI = 0.64‐1.24, *P* = 0.50, *I*^2^ = 29.2%; AG+GG vs. AA: OR = 0.91, 95%CI = 0.67‐1.23, *P* = 0.53, *I*^2^ = 58.1%; and GG vs. AA+AG: OR = 0.99, 95%CI = 0.59‐1.66, *P* = 0.50, *I*^2^ = 59.9%) (Table 3, Figure 2(a) for the G vs. A model). Moderate heterogeneity was identified in allele contrast, heterozygote model, dominant model, and recessive model. Then, metaregression analyses were conducted and they did not find any factors that might be responsible for the emergence of heterogeneity. In addition, no significant relationship was found in the subgroup analyses based on HWE status, ethnic differences, or control design (Table 3). Cumulative analyses presented a consistent trend of negative results with publication date, except for the second case-control study by Wallace et al. [25] (Figure 2(b) for the G vs. A model). Sensitivity analyses demonstrated that no single study had a significant influence on the overall outcome (Figure 2(c) for the G vs. A model). In addition, no significant asymmetrical funnel plot for publication bias was observed among the included studies, which was also demonstrated by Egger's test (G vs. A: *P* = 0.63; AG vs. AA: *P* = 0.95; GG vs. AA: *P* = 0.87; AG+GG vs. AA: *P* = 0.75; and GG vs. AA+AG: *P* = 0.86) (Figure 2(d) for the G vs. A model).

#### 3.2.2. -819T>C Polymorphism and BD Risk

**Figure 3:**
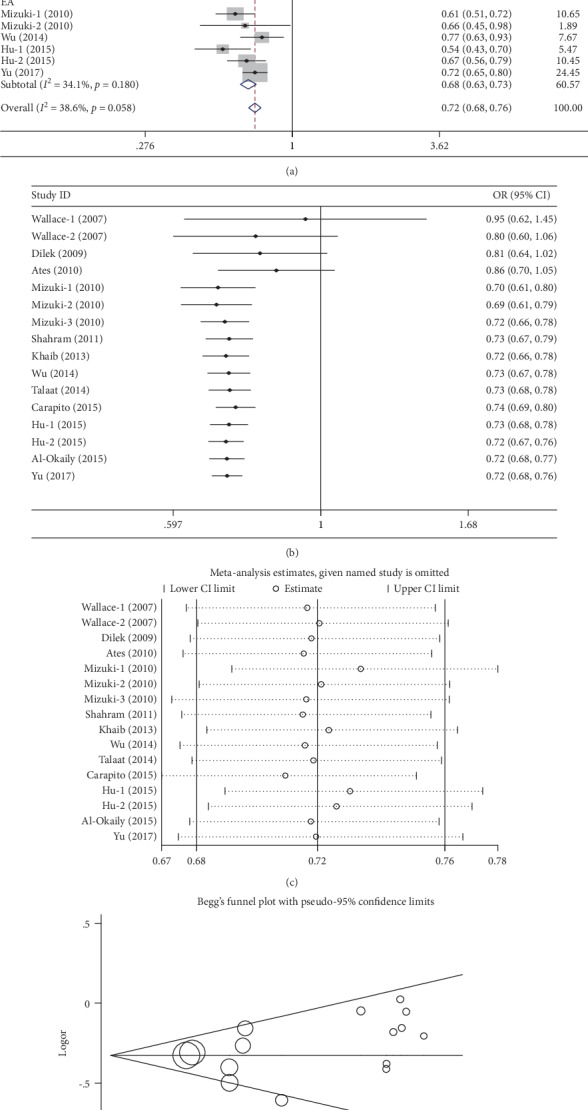
Statistical analysis of the association between IL-10 -819T>C polymorphism and BD risk in the C vs. T model. (a) ORs and 95% CIs; (b) cumulative analysis; (c) sensitivity analysis; (d) publication bias.

Sixteen case-control studies, involving 5,550 cases and 8,469 controls, were identified on the association between the -819T>C polymorphism and BD risk in this meta-analysis, and these are shown in Table 3. Overall, a significant association of protective effects was observed in all five genetic models (C vs. T, OR = 0.72, 95%CI = 0.67‐0.77, *P* < 0.01, *I*^2^ = 36.6%; TC vs. TT, OR = 0.73, 95%CI = 0.66‐0.80, *P* < 0.01, *I*^2^ = 23.0%; CC vs. TT, OR = 0.52, 95%CI = 0.39‐0.70, *P* < 0.01, *I*^2^ = 53.7%; TC+CC vs. TT, OR = 0.67, 95%CI = 0.61‐0.71, *P* < 0.01, *I*^2^ = 22.1%; and CC vs. TT+TC, OR = 0.66, 95%CI = 0.53‐0.82, *P* < 0.01, *I*^2^ = 57.8%) (Table 3, Figure 3(a) for the C vs. T model). Notably, moderate heterogeneity was identified only in the homozygote model and the recessive model; metaregression analyses were also conducted but did not find any interfering factors that might be responsible for the emergence of heterogeneity. Furthermore, significant protective associations were also found in the subgroup of the East Asians (C vs. T, OR = 0.69, 95%CI = 0.61‐0.77, *P* < 0.01, *I*^2^ = 45.2% (Figure 3(a) for the C vs. T model); TC vs. TT, OR = 0.72, 95%CI = 0.65‐0.80, *P* < 0.01, *I*^2^ = 0%; CC vs. TT, OR = 0.40, 95%CI = 0.26‐0.62, *P* < 0.01, *I*^2^ = 72.6%; TC+CC vs. TT, OR = 0.67, 95%CI = 0.60‐0.74, *P* < 0.01, *I*^2^ = 0%; and CC vs. TT+TC, OR = 0.47, 95%CI = 0.32‐0.71, *P* < 0.01, *I*^2^ = 71.1%) (Table 3). In addition, the same protective effects were also observed in both the control design groups and the genotyping groups (Table 3). Cumulative analyses steadily demonstrated a significant association with all studies (Figure 3(b) for the C vs. T model). Sensitivity analysis was conducted and did not present any apparent changes in the five genetic models (Figure 3(c) for the C vs. T model). In addition, no significant asymmetrical funnel plot for publication bias was observed among the included studies, which was also demonstrated by Egger's test (C vs. T: *P* = 0.44; TC vs. TT: *P* = 0.73; CC vs. TT: *P* = 0.94; TC+CC vs. TT: *P* = 0.87; and CC vs. TT+TC: *P* = 0.79) (Figure 3(d) for the C vs. T model).

#### 3.2.3. -592A>C Polymorphism and BD Risk

**Figure 4:**
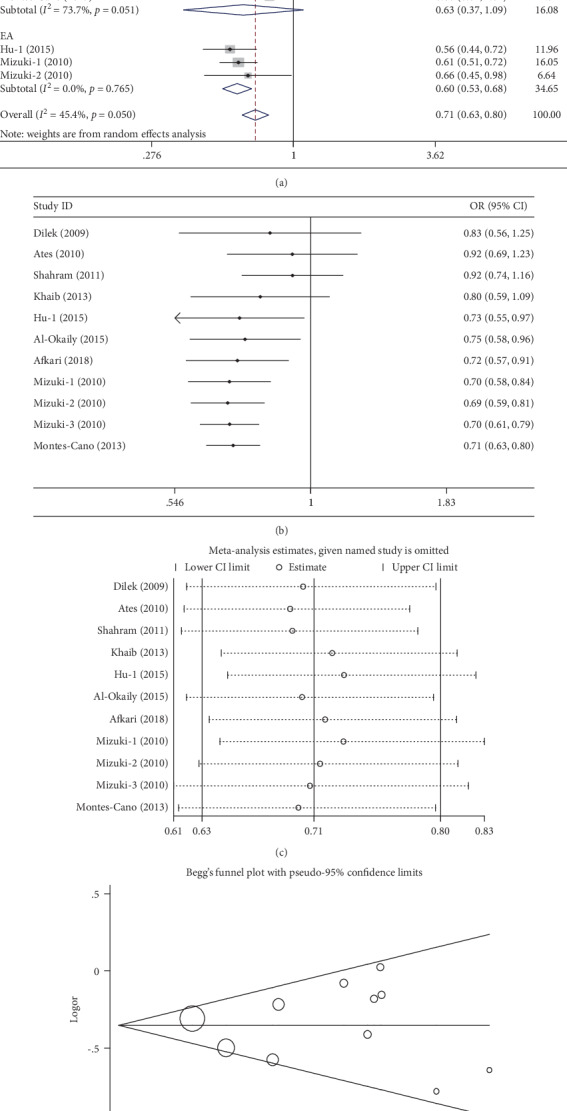
Statistical analysis of the association between IL-10 -592A>C polymorphism and BD risk in the C vs. A model. (a) ORs and 95% CIs; (b) cumulative analysis; (c) sensitivity analysis; (d) publication bias.

Eleven case-control studies, involving 3,052 cases and 3,553 controls, were identified on the association between the -592A>C polymorphism and BD risk in this meta-analysis, and these are shown in Table 3. Overall, significant protective effects were observed in four genetic models (C vs. A: OR = 0.72, 95%CI = 0.57‐0.91, *P* = 0.01, *I*^2^ = 56.5%; CC vs. AA: OR = 0.52, 95%CI = 0.28‐0.95, *P* = 0.03, *I*^2^ = 61.9%; CA+CC vs. AA: OR = 0.53, 95%CI = 0.38‐0.92, *P* = 0.02, *I*^2^ = 57.1%; and CC vs. AA+AC: OR = 0.72, 95%CI = 0.50‐0.97, *P* = 0.04, *I*^2^ = 48.0%) (Table 3, Figure 4(a) for the C vs. A model). However, the results of the stratified analysis of ethnic differences, control design, and genotyping method presented a negative association. Cumulative analyses indicated that the pooled result demonstrated a significant association between the -592A>C polymorphism and BD risk when the study of Hu et al. was added in the allele contrast and the recessive model [13], or the study of Afkari et al. was added in the homozygote model and dominant models [33] (Figure 4(b) for the C vs. A model). Sensitivity analysis was also conducted, and it demonstrated a significant association without the study by Khaib et al. [29] (Figure 4(c) for the C vs. A model). In addition, no significant asymmetrical funnel plot for publication bias was observed among the included studies, which was also demonstrated by Egger's test (C vs. A: *P* = 0.72; AC vs. AA: *P* = 0.73; CC vs. AA: *P* = 0.95; AC+CC vs. AA: *P* = 0.67; and CC vs. AA+AC: *P* = 0.40) (Figure 4(d) for the C vs. A model).

### 3.3. IL-10 Haplotype and BD Risk

Among the selected studies, only three studies provided enough data on the association between the IL-10 -1082A>G, -819T>C, and -592A>C haplotypes and BD risk. Three well-known haplotypes of ATA, ACC, and GCC were found and included in this meta-analysis. The haplotype frequency data were examined, and the quantitative synthesis did not demonstrate any significant associations when the ACC and GCC haplotypes were compared with the ATA haplotype (ACC vs. ATA: OR = 1.04, 95%CI = 0.80‐1.35, *P* = 0.78, *I*^2^ = 0%; and GCC vs. ATA: OR = 0.80, 95%CI = 0.52‐1.22, *P* = 0.30, *I*^2^ = 60.9%) (Table 4).

## 4. Discussion

To date, the pathogenesis and etiology of BD have not been elucidated. It is speculated that BD has characteristics of both inflammatory and autoimmune diseases and has a certain genetic susceptibility. Patients with BD have always suffered from serious complications, such as recurrent oral ulcers, genital ulcers, and uveitis. In addition, these microvascular inflammatory injuries are frequently involved in the gastrointestinal tract, central nervous system, large blood vessels, and limb joints in BD patients [12, 13].

The IL-10 gene is located on chromosome 1 at 1q31-32, occupies more than 4.7 kb of genomic DNA, and consists of five exons and four introns. These are the three most studied genetic variants: -1082A>G (rs1800896), -819T>C (rs1800871), and -592A>C (rs1800872). These polymorphisms and their haplotypes are associated with abnormal circulating levels of IL-10 and contribute to the pathophysiology of various autoimmune and inflammatory diseases. During the triggering and development of BD, many inflammatory markers have been proposed to be involved in the development of BD. IL-10 is one of the most important immunoregulatory cytokines and is mainly secreted by macrophage cells and other T helper 1 (Th1) and Th2 cells [38]. IL-10 participates in complex inflammatory and immune processes by promoting the widespread suppression of immune effects and the abnormal circulating level of IL-10 which has been associated with an increased risk for the development of BD.

In 2007, Wallace et al. published the first case-control study of the UK population and suggested that subjects with the -1082AA genotype were weakly associated with BD risk in the general population (OR = 1.4, 95%CI = 1.1‐1.9, *P* = 0.04), and the -819T allele also indicated an increased BD risk (OR = 1.5, 95%CI = 1.1‐2.0, *P* = 0.02). In subsequent years, an increasing number of case-control studies have been conducted to explore the association between IL-10 polymorphisms and BD susceptibility; however, the results were inconsistent and confusing. In 2013, Liang et al. published a meta-analysis on IL-10 gene polymorphisms and BD susceptibility and suggested that the IL-10 -1082A>G, -819T>C, and -592A>C polymorphisms were indeed associated with BD susceptibility [17]. However, the results were only based on two, five, and four case-controls on the IL-10 -1082A>G, -819T>C, and -592A>C loci, respectively, and the sample sizes were small as well. In 2015, Jung et al. conducted a meta-analysis on IL-10 polymorphisms and vasculitis susceptibility and reported a significant association between the IL-10 -819T>C and -592A>C polymorphisms and BD susceptibility in the stratified analysis based on only one genetic model (allele contrast). In addition, the two previous meta-analyses did not report any influence from subgroup factors, such as HWE status, ethnic differences, and the source of control groups. In 2019, two latest meta-analyses were published by Shahriyari et al. and Lee and Song. The two publications had collected all current case-control studies and made an updated conclusion on the association between IL-10 polymorphisms and BD risk [39, 40].

Today, we comprehensively summarized the current evidence and conduct this meta-analysis to explore the precise relationships between IL-10 polymorphisms and BD susceptibility with more stratified analysis. Overall, the pooled results demonstrated a significant association between the -819T>C polymorphism and BD susceptibility in all five genetic models, suggesting that the -819T>C polymorphism may decrease the risk of BD from 48% to 28%. Similarly, decreased BD risk was also observed in the subgroup analysis of the ethnic differences, control design, and genotyping method groups. In the -592A>C polymorphism, the pooled analysis of all five genetic models demonstrated that the mutation of -592A>C lowered the risk of developing BD from 48 to 29%. Moreover, similar results were also observed in many genetic models in the subgroup analysis of HWE status, ethnic differences, control design, and genotyping method groups. Regarding the -1082A>G polymorphism, no significant association was observed for BD risk, except for a few scattered cases of decreased BD risk in the G allele and the AG genotype in the EA population.

Previous studies had demonstrated that these three polymorphisms were proposed to affect the transcription efficiency and expression of the IL-10 gene [41]. More than 70% of the individual variability in human IL-10 production has been attributed to genetic variation, which could help to predict patient susceptibility to the disease as well as the severity and progression of the disease [42–44]. For the -1082A>G polymorphism, the A allele demonstrated a higher binding affinity to the transcription factor PU.1, resulting in decreased transcriptional activity and lower expression level of IL-10 [45]. In addition, the -819T>C genotype is also a functional polymorphism that could modify NF binding and result in higher levels of IL-10 production with the -819 CC genotype than with the -819 TC and TT genotypes [46]. Furthermore, the functional analysis indicated that the nucleotide mutation from cytosine (C) to adenine (A) would reduce the inhibitory effect of the transcription factor Sp1/Sp3 complex and increase the expression of IL-10 [47]. Regarding haplotype, no significant difference among the main haplotypes was observed. Due to the small sample sizes and the limited number of studies, this result requires further confirmation before being taken as fact.

To the best of our knowledge, this report describes the most comprehensive meta-analysis on the issue to date. Some advances were demonstrated in this meta-analysis. First, most case-control studies published on the main three polymorphisms were considered, which would increase the statistical power and help to increase understanding of the association between IL-10 gene polymorphisms and BD risk. Second, a stratified analysis based on HWE status, ethnic differences, control design, and genotyping methods was conducted to explore their potential relationships. Third, a more scientific retrieval strategy and statistical methodology were used, including cumulative analyses, sensitivity analyses, and haplotype analyses.

Of course, there were still some inherent limitations that should be addressed in this meta-analysis. First, there were only 7, 12, and 7 case-control studies on IL-10 -1082A>G, -819T>C, and -592A>C loci, respectively, with complete information on the wild homozygotes, heterozygotes, and mutant homozygotes; the others only had the allele data in this meta-analysis. Therefore, the synthetic results and subgroup analysis could not reveal the real association between IL-10 gene polymorphisms and BD susceptibility with limited sample size. Second, most subjects came from the ME, EA, and Europe; therefore, the results of this systematic review and meta-analysis cannot represent all ethnic populations, and the conclusions are only applicable to these ethnicities. Third, other potential factors, such as living habits, mental stress, age, and immunological disarrangement, were not assessed, as sufficient data were not available. Fourth, this meta-analysis is based on published studies; therefore, potential publication bias may be present, even if the tests utilized in this meta-analysis did not find it. Fifth, moderate or high heterogeneity could be observed in these polymorphism sites. To the best of our knowledge, clinical heterogeneity (sometimes called clinical diversity), methodological heterogeneity (sometimes called methodological diversity), and statistical heterogeneity were the three most common sources. In this meta-analysis, the severity of disease, control design, personal habits, and ethnic differences may be the potential factors that contribute to the existing heterogeneity. These above factors would lead to a deviation in the authenticity and reliability of our results. Fortunately, the heterogeneity was partly alleviated in the stratified analysis based on ethnic differences and control design. In terms of the former, the heterogeneity has obviously been relieved in the subgroup analysis of the Middle East populations in -819T>C and -592A>C polymorphism loci. Given the control design, the degree of heterogeneity has decreased in these studies with healthy-based controls in some genetic models of the -1082A>G and -819T>C polymorphisms.

## 5. Conclusions

The current evidence indicates that there is a potential association between IL-10 gene polymorphisms and BD susceptibility, especially the -819T>C and -592A>C polymorphisms. In future studies, more case-control studies with larger sample sizes and different ethnicities are needed to verify these conclusions.

## Figures and Tables

**Table 1:** Scale for quality evaluation.

Criteria	Score
Representativeness of cases	
Consecutive/randomly selected cases with clearly defined sampling frame with time, race, quantity, and defined criteria	2
Not consecutive/randomly selected case or without clearly defined sampling frame with time, race, quantity, and defined criteria	1
Not described	0
Source of controls	
Population-based	2
Hospital based or healthy based	1
Not described	0
Hardy-Weinberg equilibrium in controls	
Hardy-Weinberg equilibrium	2
Hardy-Weinberg disequilibrium	1
Not available	0
Genotyping examination	
Genotyping done under “blinded” condition and repeated again	2
Genotyping done under “blinded” condition or repeated again	1
Unblinded done or not mentioned and unrepeated	0
Subjects	
Number ≥ 300	1
Number < 300	0
Association assessment	
Assess association between genotypes and BD risk with appropriate statistics and adjustment for confounders	2
Assess association between genotypes and BD risk with appropriate statistics and without adjustment for confounders	1
Inappropriate statistics used	0

**Table 2:** Characteristics of included studies on IL-10 gene polymorphisms and Behcet's disease risk.

First author	Year	Country/region	Racial	Source of controls	Case	Control	Genotype distribution						Genotyping methods	*P* for HWE	NOS
							Case			Control					
							-1082A>G (rs1800896)								
							AA	AG	GG	AA	AG	GG			
Wallace-1	2007	UK	Europe	HB	63	182	27	23	13	59	75	48	PCR-SSP	0.02	5
Wallace-2	2007	Jordan and Palestinian	ME	HB	115	113	50	50	15	46	40	27	PCR-SSP	<0.01	5
Dilek	2009	Turkey	ME	PB	96	124	26	66	4	56	58	10	PCR-SSP	0.35	8
Ates	2010	Turkey	ME	PB	102	102	39	50	13	44	50	8	ARMS-PCR	0.23	7
Shahram	2011	*Iran*	ME	HB	147	140	58	81	8	53	75	12	PCR-SSP	0.04	5
Talaat	2014	Egypt	ME	HB	87	97	32	34	21	32	53	12	MS-PCR	0.16	6
Hu-1	2015	China	EA	PB	300	350	271	29	0	293	56	1	PCR-RFLP	0.32	10
Al-Okaily	2015	Saudi Arabia	ME	PB	61	211	14	37	10	36	159	16	ARMS-PCR	<0.01	7
Barış	2016	Turkey	ME	HB	71	70	98^a^	44^b^		99^a^	41^b^		PCR-RFLP	NA	4
							-819T>C (rs1800871)								
							TT	TC	CC	TT	TC	CC			
Wallace-1	2007	UK	Europe	HB	63	182	7	28	44	13	67	102	PCR-SSP	0.66	6
Wallace-2	2007	Jordan and Palestinian	ME	HB	115	113	11	61	43	10	43	60	PCR-SSP	0.57	6
Dilek	2009	Turkey	ME	PB	96	124	7	50	39	13	47	64	PCR-SSP	0.33	8
Ates	2010	Turkey	ME	PB	102	102	10	45	47	11	44	47	ARMS-PCR	0.88	7
Mizuki-1	2010	Japan	EA	HB	611	737	935^c^	287^d^		979^c^	495^d^		GeneChip	NA	5
Mizuki-2	2010	Korea	EA	HB	119	140	184^c^	54^d^		194^c^	86^d^		GeneChip	NA	4
Mizuki-3	2010	Turkey	ME	HB	1215	1279	931^c^	1499^d^		801^c^	1757^d^		GeneChip	NA	5
Shahram	2011	Iran	ME	HB	147	140	12	64	71	12	57	71	PCR-SSP	0.91	6
Khaib	2013	Algeria	Europe	PB	51	96	11	23	17	5	41	50	Direct sequencing	0.35	7
Wu	2014	China	EA	PB	407	679	209	167	31	302	295	82	Sequenom MassArray	0.45	9
Talaat	2014	Egypt	ME	HB	87	97	10	35	42	6	41	50	MS-PCR	0.53	6
Carapito	2015	Iran	ME	HB	552	341	376^c^	728^d^		255^c^	579^d^		Taqman	NA	5
Hu-1	2015	China	EA	PB	300	350	168	126	6	146	160	44	PCR-RFLP	0.99	10
Hu-2	2015	China	EA	PB	418	1403	216	175	27	558	679	166	PCR-RFLP	0.06	10
Al-Okaily	2015	Saudi Arabia	ME	PB	61	211	12	22	27	21	102	88	ARMS-PCR	0.27	8
Yu	2017	China	EA	PB	1206	2475	661	459	86	1121	1078	276	Real-time PCR	0.49	9
							-592A>C (rs1800872)								
							AA	AC	CC	AA	AC	CC			
Dilek	2009	Turkey	ME	PB	96	124	7	50	39	13	47	64	PCR-SSP	0.33	8
Ates	2010	Turkey	ME	PB	102	102	10	45	47	11	44	47	ARMS-PCR	0.88	7
Mizuki-1	2010	Japan	EA	HB	611	737	935^e^	287^f^		979^e^	495^f^		GeneChip	NA	5
Mizuki-2	2010	Korea	EA	HB	119	140	184^e^	54^f^		194^e^	86^f^		GeneChip	NA	4
Mizuki-3	2010	Turkey	ME	HB	1215	1279	931^e^	1499^f^		801^e^	1757^f^		GeneChip	NA	5
Shahram	2011	*Iran*	ME	HB	147	140	12	66	69	12	57	71	PCR-SSP	0.91	6
Khaib	2013	Algeria	Europe	PB	51	96	11	23	17	5	41	50	Direct sequencing	0.35	7
Montes-Cano	2013	Spain	Europe	PB	304	313	172^e^	408^f^		157^e^	463^f^		PCR-SSOP	NA	6
Hu-1	2015	China	EA	PB	300	350	170	120	10	140	174	36	PCR-RFLP	0.09	10
Al-Okaily	2015	Saudi Arabia	ME	PB	61	211	12	22	27	21	102	88	ARMS-PCR	0.27	8
Afkari	2018	Iran	ME	HB	46	61	24	20	2	16	41	4	PCR-RFLP	<0.01	5

HWE in control. PB: hospital-based control; HB: healthy-based control/normal control; a: A allele; b: G allele; c: T allele; d: C allele; e: A allele; f: C allele; EA: East Asian; ME: Middle East.

**Table 3:** Summary ORs and 95% CI of IL-10 gene polymorphisms and Behcet's disease risk.

Locus	N^∗^	OR	95% CI	*P*	*I* ^2^ (%)^a^	OR	95% CI	*P*	*I* ^2^ (%)^a^	OR	95% CI	*P*	*I* ^2^ (%)^a^	OR	95% CI	*P*	*I* ^2^ (%)^a^	OR	95% CI	*P*	*I* ^2^ (%)^a^
-1082A>G (rs1800870)		G vs. A				AG vs. AA				GG vs. AA				AG+GG vs. AA				GG vs. AA+AG			
Total	9	0.95	0.79-1.15	0.60	46.1	0.91	0.65-1.29	0.60	64.2	0.89	0.64-1.24	0.50	29.2	0.91	0.67-1.23	0.53	58.1	0.99	0.59-1.66	0.50	59.9
HWE—yes	4	1.04	0.72-1.54	0.83	69.1	0.87	0.65-1.17	0.99	81.9	1.45	0.83-2.51	0.19	0	1.05	0.58-1.92	0.87	79.0	1.30	0.61-2.77	0.59	41.6
HWE—no	4	0.85	0.70-1.03	0.09	0	0.99	0.51-1.94	0.36	0	0.68	0.44-1.03	0.07	15.8	0.81	0.61-1.07	0.13	0	0.83	0.42-1.63	0.95	66.4
Ethnicity																					
Europe	1	0.72	0.48-1.09	0.12	NA	0.67	0.35-1.29	0.23	NA	0.59	0.28-1.27	0.18	NA	0.64	0.36-1.15	0.14	NA	0.73	0.36-1.45	0.37	NA
ME	7	1.05	0.90-1.22	0.55	7.5	1.05	0.71-1.55	0.79	61.1	1.00	0.69-1.45	1.00	38.3	1.06	0.77-1.45	0.72	46.3	1.07	0.57-2.04	0.83	69.0
EA	1	0.56	0.35-0.89	0.01	NA	0.56	0.35-0.90	0.02	NA	0.36	0.01-8.88	0.53	NA	0.55	0.34-0.89	0.01	NA	0.39	0.02-9.55	0.56	NA
Design																					
HB	5	0.89	0.74-1.07	0.21	4.0	0.87	0.65-1.17	0.36	0	0.74	0.43-1.28	0.28	42.9	0.84	0.64-1.09	0.19	0	0.83	0.42-1.63	0.59	67.7
PB	4	1.02	0.70-1.47	0.93	68.3	0.98	0.49-1.95	0.96	82.1	1.37	0.76-2.46	0.30	0	1.01	0.53-1.90	0.98	80	1.30	0.59-2.86	0.52	43.2
-819T>C (rs1800871)		C vs. T				TC vs. TT				CC vs. TT				TC+CC vs. TT				CC vs. TT+TC			
Total	16	0.72	0.68-0.76	<0.01	38.6	0.73	0.66-0.80	<0.01	23.0	0.52	0.39-0.70	<0.01	53.7	0.67	0.61-0.71	<0.01	22.1	0.66	0.53-0.82	<0.01	57.8
Ethnicity																					
Europe	2	0.67	0.33-1.36	0.27	78.6	0.46	0.16-1.37	0.17	49.3	0.36	0.07-1.83	0.22	77.0	0.41	0.11-1.58	0.20	70.1	0.70	0.33-1.47	0.35	65.0
ME	8	0.79	0.72-0.86	<0.01	0	0.92	0.55-1.52	0.74	40.8	0.78	0.53-1.14	0.20	0	0.84	0.59-1.21	0.36	7.4	0.81	0.65-1.01	0.06	5.2
EA	6	0.68	0.63-0.73	<0.01	34.1	0.72	0.65-0.80	<0.01	0	0.40	0.26-0.62	<0.01	72.6	0.67	0.60-.74	<0.01	0	0.47	0.32-0.71	<0.01	71.1
Design																					
HB	8	0.74	0.68-0.79	<0.01	36.7	0.92	0.56-1.49	0.73	0	0.74	0.46-1.20	0.23	0	0.83	0.52-1.21	0.42	0	0.81	0.62-1.04	0.10	10.7
PB	8	0.71	0.63-0.80	<0.01	45.7	0.72	0.65-0.80	<0.01	39.0	0.46	0.32-0.67	<0.01	65.4	0.66	0.56-0.78	<0.01	42.3	0.59	0.44-0.80	<0.01	64.1
Genotyping																					
PCR	9	0.76	0.66-0.89	<0.01	42.3	0.72	0.61-0.84	<0.01	22.8	0.57	0.37-0.89	0.01	59.8	0.64	0.55-0.75	<0.01	3.8	0.69	0.50-0.94	0.02	66.6
Other	7	0.71	0.65-0.78	<0.01	43.2	0.73	0.57-0.92	0.01	47.9	0.47	0.32-0.71	<0.01	50.2	0.67	0.50-0.88	<0.01	62.2	0.59	0.48-0.73	<0.01	0
-592A>C (rs1800872)		C vs. A				AC vs. AA				CC vs. AA				AC+CC vs. AA				CC vs. AA+AC			
Total	11	0.71	0.63-0.80	<0.01	45.4	0.64	0.40-1.02	0.06	59.2	0.52	0.28-0.95	0.03	61.9	0.59	0.38-0.92	0.02	57.1	0.70	0.50-0.97	0.04	48.0
HWE—yes	6	0.75	0.58-0.96	0.02	60.9	0.71	0.43-1.19	0.20	59.6	0.54	0.28-1.04	0.07	67.8	0.65	0.40-1.06	0.08	59.0	0.69	0.48-1.00	0.05	56.6
HWE—no	1	0.53	0.29-0.95	0.03	NA	0.33	0.14-0.74	0.01	NA	0.33	0.05-2.04	0.24	NA	0.33	0.14-0.73	0.01	NA	0.65	0.11-3.70	0.63	NA
Ethnicity																					
Europe	2	0.63	0.37-1.09	0.10	73.7	0.25	0.08-0.82	0.02	NA	0.15	0.05-0.51	<0.01	NA	0.20	0.07-0.61	0.01	NA	0.46	0.23-0.93	0.03	NA
ME	6	0.77	0.69-0.85	<0.01	7.6	0.77	0.39-1.53	0.45	65.7	0.82	0.53-1.27	0.37	0	0.74	0.42-1.30	0.30	55.3	0.87	0.64-1.13	0.29	0
EA	3	0.60	0.53-0.68	<0.01	0	0.57	0.41-0.78	<0.01	NA	0.23	0.11-0.48	<0.01	NA	0.51	0.37-0.70	<0.01	NA	0.30	0.15-0.62	<0.01	NA
Design																					
HB	5	0.70	0.64-0.76	<0.01	39.1	0.61	0.18-2.11	0.43	76.6	0.79	0.36-1.71	0.55	8.4	0.58	0.18-1.85	0.36	74.4	0.84	0.54-1.32	0.45	0
PB	6	0.73	0.59-0.91	0.01	57.2	0.65	0.37-1.16	0.14	61.6	0.48	0.23-1.01	0.05	69.7	0.60	0.35-1.02	0.06	60.3	0.65	0.42-1.02	0.06	63.2
Genotyping																					
PCR	7	0.77	0.64-0.93	0.01	46.1	0.70	0.43-1.14	0.16	60.2	0.62	0.34-1.11	0.11	56.2	0.66	0.43-1.01	0.05	53.1	0.74	0.52-1.06	0.10	48.6
Other	4	0.65	0.56-0.76	<0.01	47.4	0.25	0.08-0.82	0.02	NA	0.15	0.05-0.51	<0.01	NA	0.20	0.07-0.61	0.01	NA	0.46	0.23-0.93	0.03	NA

^∗^Numbers of comparisons. ^a^Test for heterogeneity. ME: Middle East; EA: East Asia; PB: population based; HB: healthy-based control/normal control.

**Table 4:** Meta-analysis of the IL-10 -1082A>G, -819T>C and -592A>C haplotype and BD risk.

Contrast		OR	95% CI	*P*	*I* ^2^	*P*_Egger's test_
ACC vs. ATA	Total	1.04	0.80-1.35	0.78	0	0.11
	Turkey	1.12	0.81-1.57	0.45	0	
	Iran	0.92	0.60-1.39	0.68	NA	
GCC vs. ATA	Total	0.80	0.52-1.22	0.30	60.9	0.41
	Turkey	0.67	0.40-1.13	0.14	55.9	
	Iran	1.07	0.71-1.61	0.74	NA	

NA: not available.

## Data Availability

The raw data supporting this meta-analysis are from previously reported studies and datasets. All included data are also available from the corresponding author upon request.

## Supplementary Materials

Supplementary Materials Search strategies for the online databases.
