# Supplementary material for: Association between Interleukin-10 Gene Polymorphisms and Behcet's Disease Susceptibility: Evidence from a Meta-Analysis
Source: J Immunol Res. 2020 Jun 19;2020:9382609. doi: 10.1155/2020/9382609 (PMC7321531; doi:10.1155/2020/9382609)
Supplement: Supplementary Materials — Search strategies for the online databases. [file 9382609.f1.docx]

Search strategies in the online databases

| Online databases | Search strategy |
| --- | --- |
| Pubmed | ((IL-10) OR (Interleukin-10) OR (Interleukin 10) OR rs1800896 OR rs1800871 OR rs1800872) AND (polymorphism OR variant OR mutation) AND ((Behcet's disease) OR BD) |
| EMBASE | ('interleukin 10' OR 'il 10' OR rs1800896 OR rs1800871 OR rs1800872) AND ('polymorphism' OR 'variant' OR 'mutation') AND ('Behcets disease' OR BD) |
| Websci | (TS=((Interleukin 10) OR (IL-10) OR (rs1800896) OR (rs1800871) OR (rs1800872))) AND (TS=((polymorphism) OR (variant) OR (mutation))) AND (TS=((Behcet's disease) OR (BD))) |
| CNKI | SU=('多态性'+'变异'+'等位基因'+'突变'+'基因型')*('白介素10'+'白细胞介素10'+ 'rs1800896'+'rs1800871'+'rs1800872')*( '白塞氏病') +'Behcet's disease') |
| Wanfang | (主题:(多态性)+主题:(变异)+主题:(等位基因)+主题:(突变)+主题:(基因型))* (主题: (IL-10)+主题:( Interleukin 10)+主题:( Interleukin 10) +主题: (白介素10)+ 主题: (白细胞介素10)+ 主题: (rs1800896)+ 主题: (rs1800871)+ 主题: (rs1800872))* (主题:( 白塞氏病)+主题:( Behcet's disease)) |
